# Spatially coincident vibrotactile noise improves subthreshold stimulus detection

**DOI:** 10.1371/journal.pone.0186932

**Published:** 2017-11-01

**Authors:** Luis T. Arredondo, Claudio A. Perez

**Affiliations:** 1 Department of Electrical Engineering, Universidad de Chile, Santiago, Chile; 2 Department of Electrical Engineering and Advanced Mining Technology Center, Universidad de Chile, Santiago, Chile; University of Melbourne, AUSTRALIA

## Abstract

Stochastic Resonance (SR) is a phenomenon, mainly present in nonlinear detection systems, in which the addition of certain amount of noise, called optimal noise, has proven to enhance detection performance of subthreshold stimuli. When added noise is present only during the stimulus, an additional enhancement can be reached. This phenomenon was called time Coincidence Enhanced Stochastic Resonance (CESR). The aim of this study was to study the effect of spatially distributed vibrotactile noise in subthreshold stimuli detection. The correct response rates from two different stimuli conditions were compared, using four tactile stimulator systems to excite four different spatial locations on the fingertip. Under two different conditions, the stimuli were present in only one randomly chosen stimulator. For the first condition, all stimulators contain optimal noise level. In the second condition, the optimal noise was present only at the stimulator with the stimulus. SR threshold principle should not produce different correct response rates between the two conditions, since in both cases the noise enables the subthreshold stimulus to go above threshold. The stimulus signal used was a rectangular displacement controlled pulse that lasted 300ms within a 1.5s attention interval, applied to the exploratory zone of the index finger of 13 human subjects. For all subjects it was found that detection rates were better (p<0.0003) when noise was spatially coincident with the stimulus, compared to the condition in which noise was present simultaneously in all the stimulators. According to our literature review this is the first report of SR being influenced by the spatial location of the noise. These results were not found previously reported, so represent the discovery of a new phenomenon. We call this phenomenon Spatial-Coincidence-Enhanced Stochastic Resonance (SCESR). As results show, the optimal noise level is dependent on the relative position between stimulus and noise.

## Introduction

Stochastic resonance (SR) is a counterintuitive phenomenon in which the addition of certain amount of noise increases detection of weak signals in some nonlinear systems such as touch [1], [2], [3], [4], [5], [6]. There is multiple experimental evidence that SR occurs in the human tactile system [2], [3], [7], [8], [9]. In that experimental evidence, authors used a subthreshold mechanical stimulus in the form of pulse and mechanical noise in the form of random vibrations. They demonstrated that true positive detection rates were higher for certain levels of noise added to the subthreshold stimuli signal. During the last years, SR has been demonstrated in the human tactile system, in fingertips, soles of the feet, lips, tongue, and many other glabrous skin regions of the human body and in other senses as well [8], [10], [11], [12], [13]. SR phenomena are commonly explained assuming that noise works as a pedestal allowing weak signals to exceed sensory thresholds [2], [8], [10].

Perez et al. [1] performed an experiment using a pulse type mechanical stimulus, with amplitude below tactile threshold and optimal mechanical noise, which produced SR, under two different conditions. In the first condition, noise was present throughout the attention interval; in the second condition, noise was present only during the stimulus time interval. Previous SR studies found no difference for these two conditions [1]; however, the authors found that detection rates were higher when the noise was synchronized with the stimulus. As a possible explanation for this novel phenomenon, the authors postulated in [1] that noise coincident with the stimuli might reduce Temporal Uncertainty (TU), according to the sensorial model proposed by Tanner [14]. The sensory detection enhancement produced by temporal coincidence between stimuli and noise raises the question of whether a similar phenomenon would be present for spatially coincident stimulus and noise. Spatially coincident noise might help to reduce Spatial Uncertainty (SU) and therefore expect enhanced detection for this new condition.

The objective of the present study is to test predictions made from the SR model under two different conditions: In the first condition, the noise was applied at the stimulus location and at the other three locations, and the other with the noise coincident at the same spatial location as that of the rectangular pulse stimulus. By using a four mechanical stimulator system in the first condition, the stimulus is present in only one of the four stimulators, while noise is present in the four stimulators simultaneously during the whole attention interval. In the second condition, the noise is present only at the same position as the stimulus. Since the use of optimal noise would help subthreshold stimuli to exceed sensory detection level [2] in both conditions, SR theory does not predict differences between the two conditions. Nevertheless, our results show that positive detection rates are higher in the second condition when noise is spatially coincident with the stimulus, compared to the first condition in which noise is present in all the stimulators.

Although there are some previous studies on the effects of SU [15], [16], [17], on subthreshold stimuli detection, they focus on visual perception and not on the human tactile system. Therefore, our proposed study is novel according to our literature review.

Analysis of the relation between SR and SU could contribute to explain the results reached in this study and help explore new phenomena that have not been predicted by SR. Additionally, the study of this phenomenon could lead to a better understanding of human tactile perception, leading to improvements in haptic interface development, diagnostic tools, prosthesis design, and human mimetic transducers.

## Materials and methods

### Subjects

A total of 13 subjects, 1 female and 12 male, with ages between 22 and 39 years participated in our experiment. Each one of them had read and signed an informed consent form, which describes the characteristics of the experiment, the purpose of the investigation, and a statement indicating that there was no risk to participants. Only persons that declared the absence of diagnosed neurological conditions were able to participate. There was a training process for subjects participating in the study. The experiments were properly approved as states the resolution No.11 from June 14, 2006, by the Bioethics Committee from INTA institute, Universidad de Chile.

**Fig 1:**
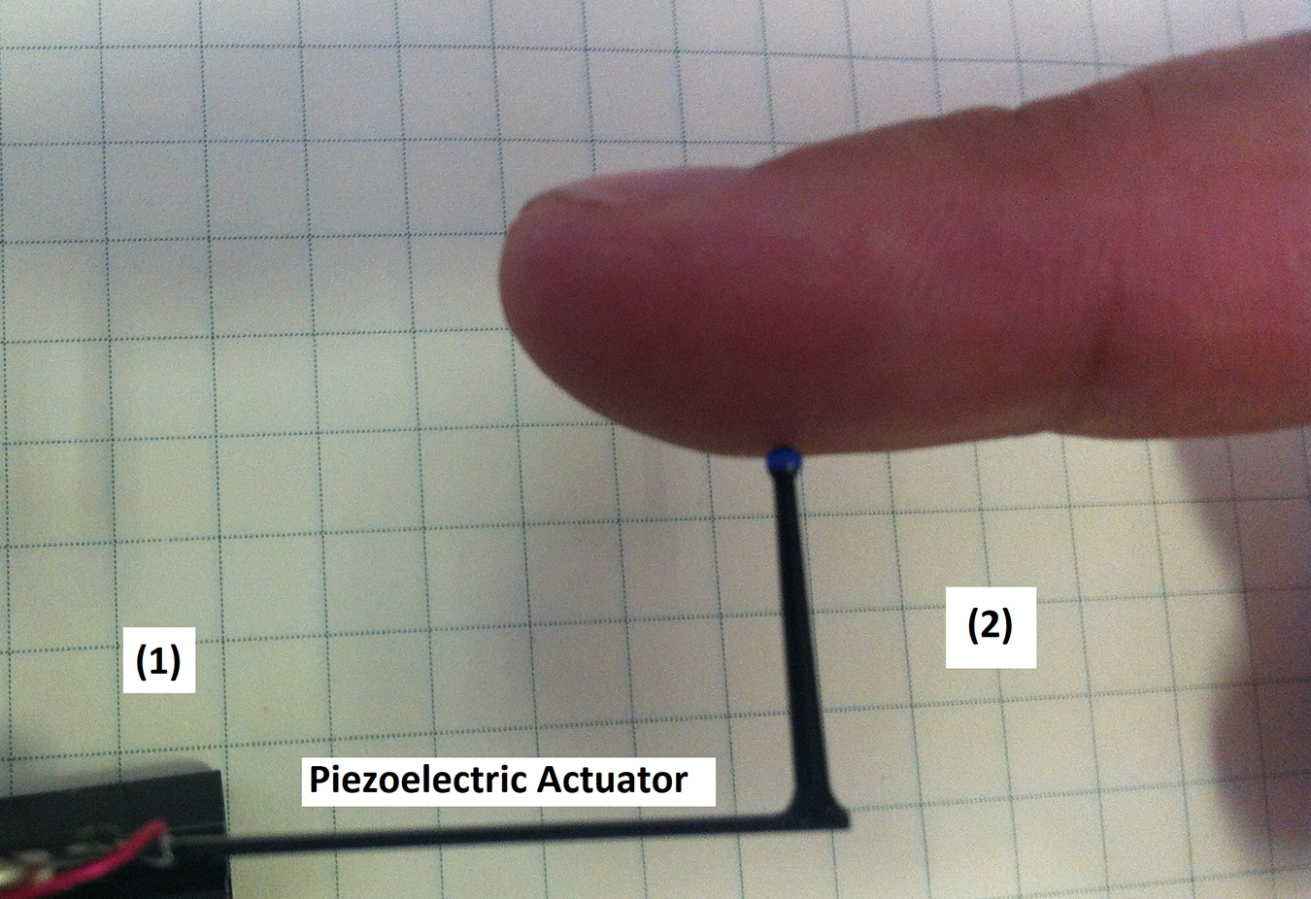
Photograph that shows a piezoelectric actuator used for mechanical stimulation. (1) Connection electrodes attached to piezo electric lead terminals, (2) a round ended plastic tip fixed to one end of the piezoelectric stripe. This picture shows only one of the four mechanical stimulators.

Four piezoelectric stripe actuators (APC 40–1035, APC International, Ltd.), were used as mechanical stimulators to excite the testing area of the index finger. A 1.5mm diameter round-ended plastic tip, similar to the one used by Collins in [2], was attached to the end of each actuator, mounted in a cantilever manner as is shown in Fig 1. In this way, the piezoelectric stripe bends when a voltage is applied to its contacts, resulting in mechanical stimulation of the fingertip.

**Fig 2:**
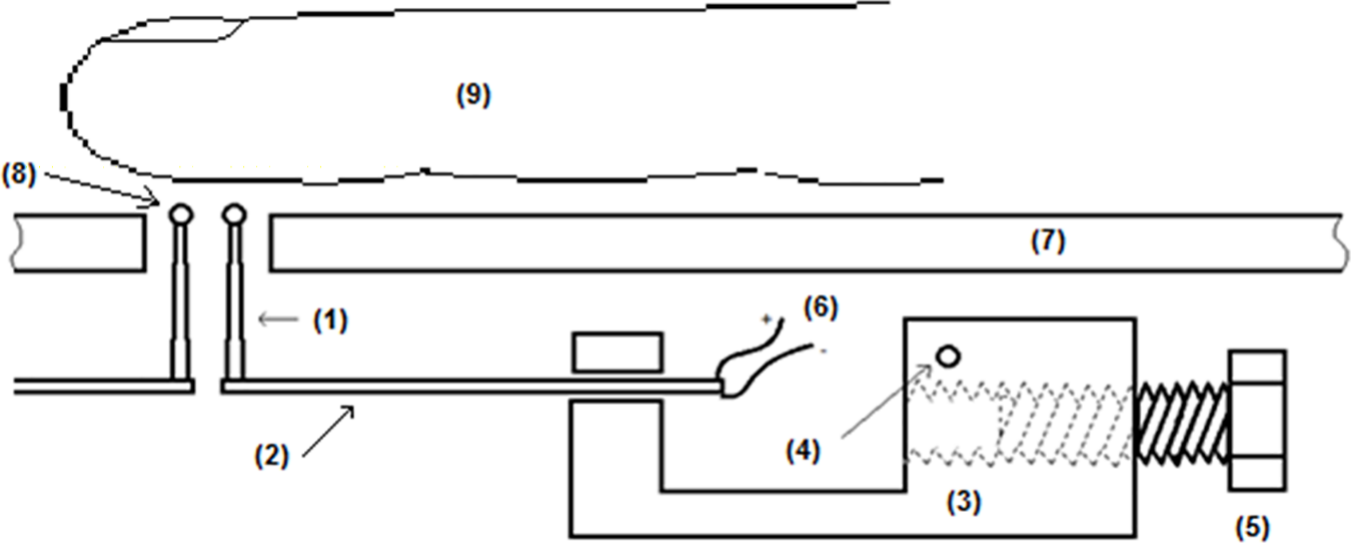
Schematic diagram of the mechanical stimulation module. (1) Plastic tip, (2) piezoelectric stripe actuator, (3) rocker metallic mounting, (4) pivoting axis, (5) counterweight bolt, (6) actuator electrode, (7) upper surface where subjects rest their fingers and hands, (8) round plastic ends in contact with the fingertip, (9) a subject’s index finger. This Fig shows only two of the four mechanical stimulator modules.

**Fig 3:**
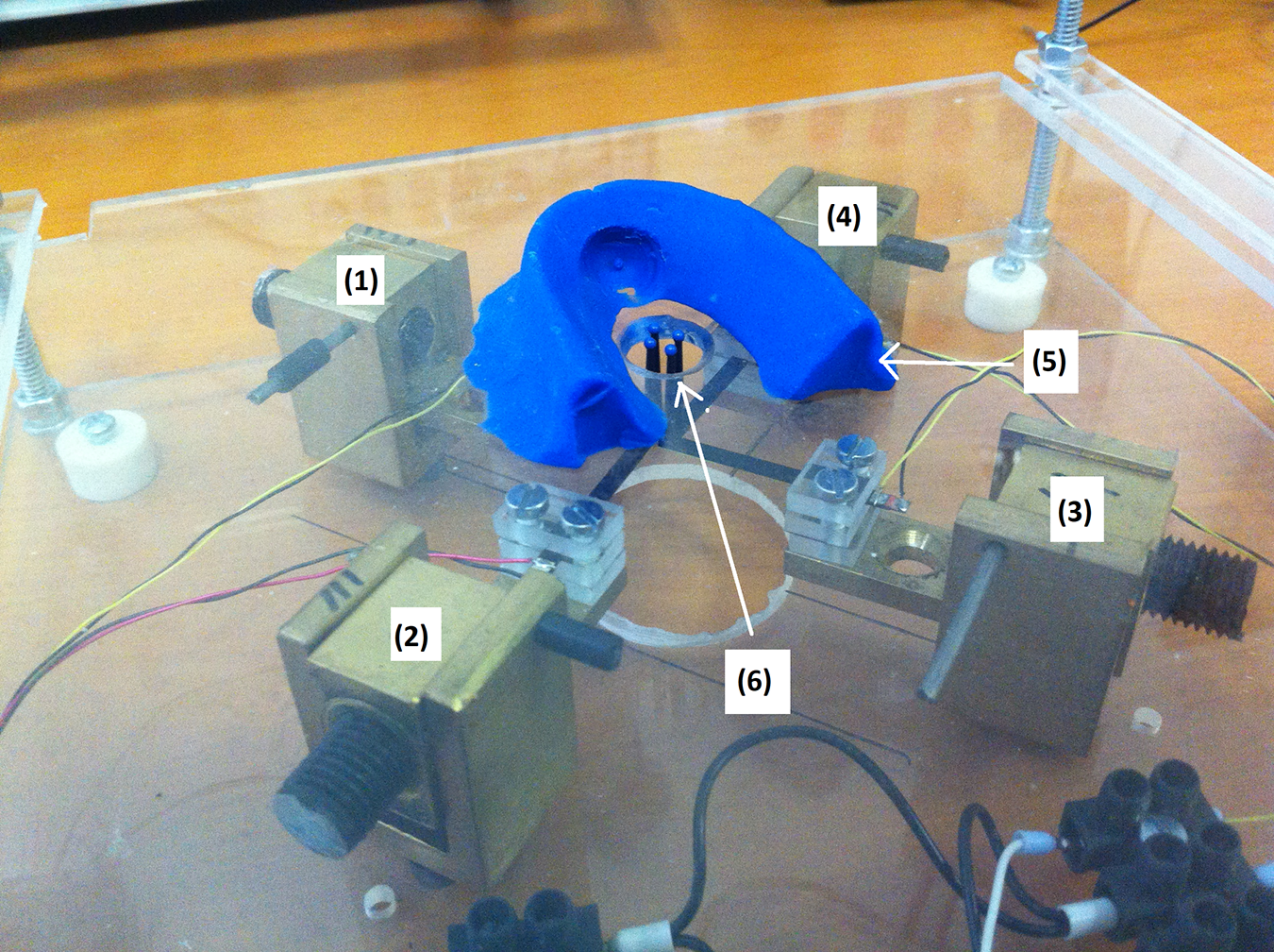
Actual four channel mechanical stimulation system. (1), (2), (3) and (4) mechanical stimulation modules. (5) Plastic clay that aids subjects to place their index finger tips over the stimulators, (6) set of 4 round ended plastic tips that make contact with the index fingertip.

Each piezoelectric stripe is mounted on a rocker piece made of metal as shown in Fig 2. A bolt acting as a counterweight was included allowing adjustment of a 200 mN static force applied to the skin by each individual stimulator. A picture of the four mechanical stimulators mounted for the experiment can be observed in Fig 3. The four stimulator tips were arranged, as shown in Fig 3, following a square distribution with a 3 mm edge-to-edge separation among them, so they would stimulate different receptive fields of Meissner mechanoreceptors on the fingertip [18], [19].

In order to produce mechanical stimulation, each piezoelectric stripe must be driven by a voltage level. The piezoelectric stripe bends up or down depending on the electric polarity of the voltage applied. The piezoelectric vertical displacement is determined by the amplitude of the applied voltage. An optical sensor (MTI 1000 Fotonic Sensor, MTI instruments) was used to precisely determine the magnitude of the vertical displacement as a function of the voltage applied to the piezoelectric actuator, and a linear relationship between the stimulator displacement and the applied voltage–which the manufacturer specified- was verified.

A four channel USB digital-to-analog converter (NI9263 16bit, +/-10V, 100KS/s, National Instruments) was used to generate the voltage levels applied to each piezoelectric stripe. A 30Hz fourth-order Butterworth low pass filter was used on each channel, to limit stimulation to the non-Pacinian channels [18], [19]. Finally a power amplifier able to generate up to +/-40[V] drove all four piezoelectric mechanical stimulators.

### Measurements

**Fig 4:**
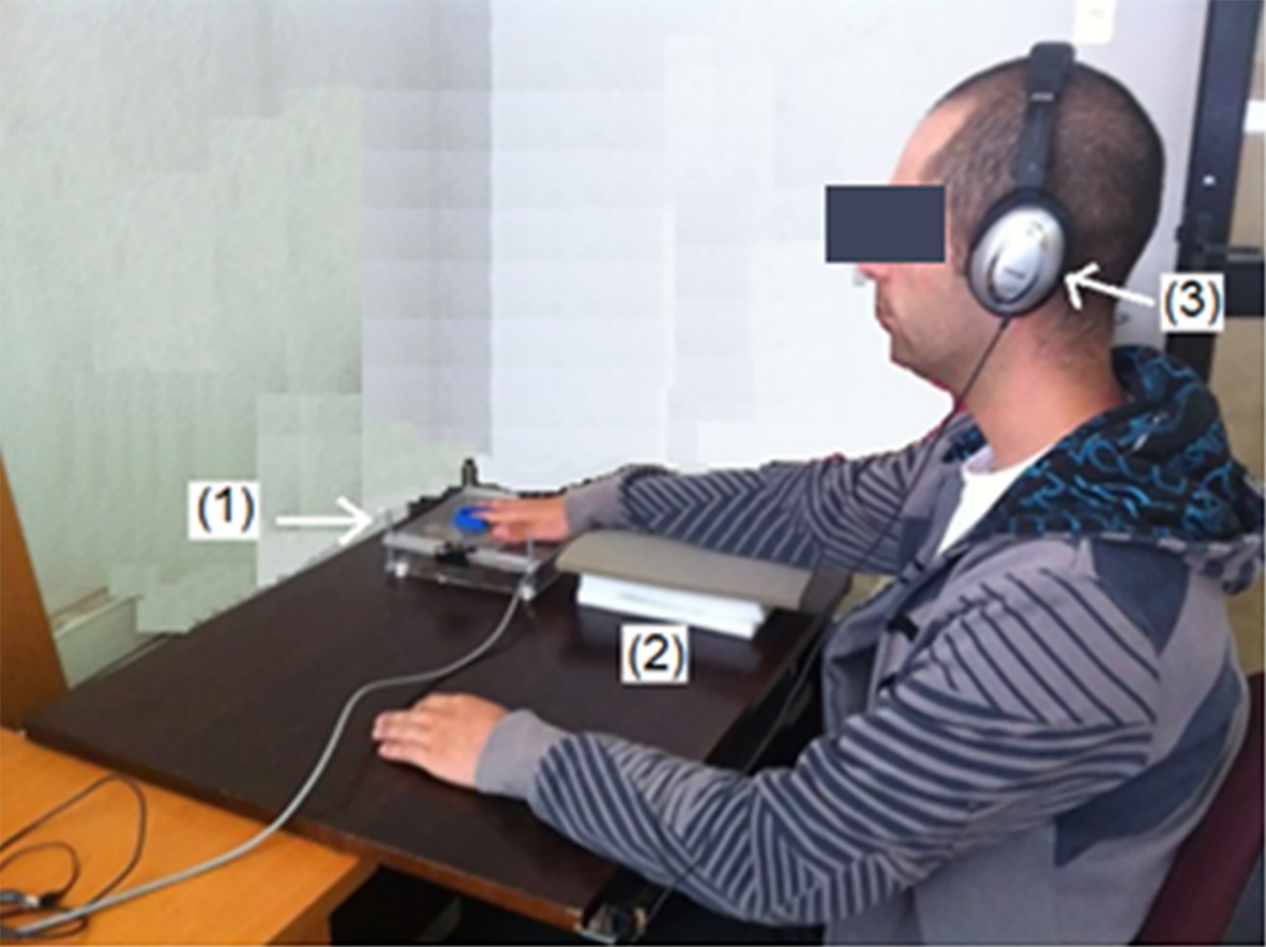
Actual scene from a subject under study. (1) The hand support, subjects lay their hands on a flat surface so that their index fingers are located directly over the mechanical stimulators, (2) an arm support, a set of foam blocks provides comfortable support during the experiment procedures, (3) headphones through which subjects receive audible cues for the start of the attention interval during which the stimulus may occur.

Each subject remained seated in front of a table as seen in Fig 4, resting their right arm over a synthetic foam support. The subjects were required to lay their right hands on a flat surface on top of the structure that contains the mechanical actuators. As shown in Figs 3 and 4, a piece of blue clay helped to maintain the index finger in place, touching the plastic tips of the actuators. Subjects wore headphones to avoid distractions and to receive an auditory cue for the start of the attention interval, which lasted 1.5s. The individual who appears on this manuscript has properly given written informed consent (as outlined in PLOS consent form) to publish these case details, including his picture shown in Fig 4.

#### Experiments

Three experiments were performed on each subject: perception thresholds, optimal noise, and noise spatial distribution assessment. The objective of experiment 1 is to determine tactile perception thresholds for: rectangular shape stimuli, noise stimuli present in one stimulation point, and noise stimuli present in four stimulation points. The objective of experiment 2 is to determine the optimal level of noise that produces stochastic resonance for two noise spatial distributions: noise only in the same stimulator that contains the rectangular shape stimuli and noise in all four stimulators simultaneously. The objective of experiment 3 is to determine which noise spatial distribution, that produces stochastic resonance, achieves higher correct detection rates on subjects. On each experimental session all three experiments were consecutively performed to a single subject.

**Fig 5:**
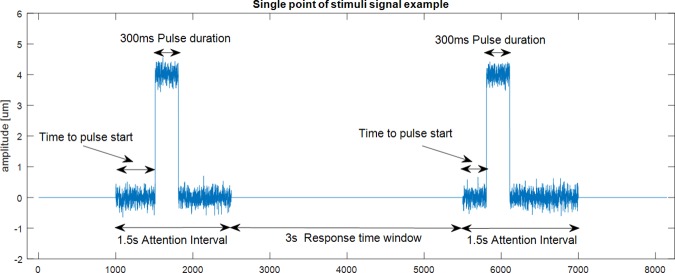
Two consecutive trials example for single channel stimuli signal used in experiments. Noise last during all attention interval. Pulse signal has a fixed duration of 300ms. Time to pulse start is randomly selected from trial to trial. A 3s pause between trials with no stimulus is used as response window.

All performed experiments have the same protocol structure: an attention interval of 1.5s which contains the stimulus signals, followed by a 3s response window before next trial. Fig 5 shows an example of this protocol for a single point of stimulation, and two consecutives trials.

#### Experiment 1

The objective of this experiment is to determine the tactile sensation threshold for each subject. Subsequent sub-threshold stimulation depends on the values determined in this experiment. Three perception thresholds are estimated for the rectangular pulse and two spatial distributions of the Gaussian noise stimulus. In all cases thresholds are measured using a yes/no task [20] with catch trials. The stimulus level is adjusted in each trial using the staircase method [20], [21]. The amplitude is controlled for the pulse stimulus, and the standard deviation for noise is added to the stimulus. After some trials the stimulus amplitude should correspond to threshold, where 50% correct detection occurs [20]. Each threshold level is recorded.

#### Experiment 2

The aim of this experiment is to determine the two optimal noise levels that produce stochastic resonance [22], for two spatial distributions, on the first case the noise is added only at the same stimulator that contains the stimulus signal, while on the other case the noise is added on all four stimulators, therefore two optimal level noises are determined, one for each noise spatial distribution. In both cases the stimulus pulse was allocated randomly to one of four stimulators as shown in Fig 3. The pulse amplitude was 80% of the previously measured pulse threshold. Five different noise levels were used, with standard deviation values of 0%, 30%, 60%, 90%, and 120% of those previously measured noise thresholds. In one case, noise was added to the stimulus signal at the same location as the stimulus signal. In the other case, noise was added simultaneously to all four stimulators, not just to the one that carried the pulse stimulus. In both cases, noise was present during the whole 1.5s attention interval. A yes/no task [20] was performed for each case and the percentage of correct responses, P(C), was computed from a total of 40 trials for every single noise level. Half of the trials contained the stimulus signal plus noise and the other half contained only noise. Therefore, a correct response occurred when the subject’s answer was ‘yes’ when the stimulus was present, and when the subject’s answer is ‘no’ and the stimulus was not present. The noise level that produced the highest P(C), stochastic resonance point [2], for each case was recorded.

**Fig 6:**
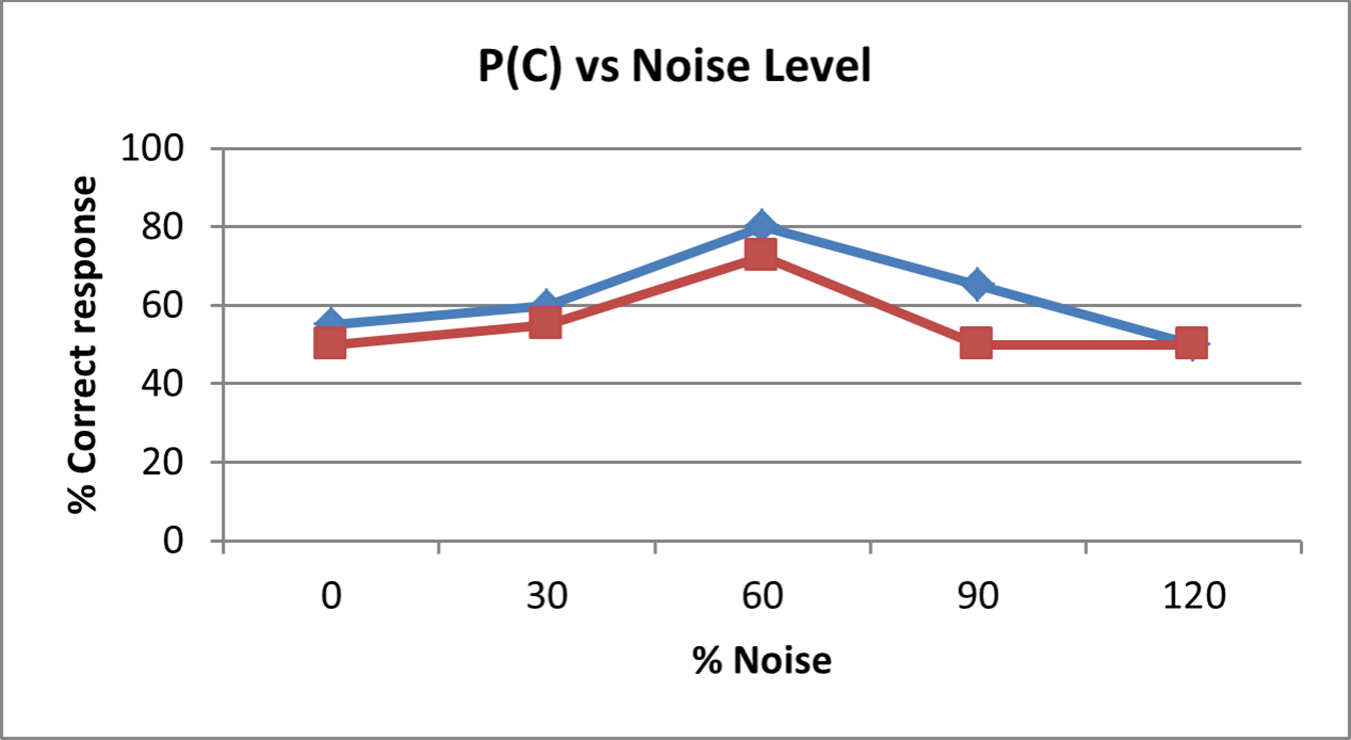
Percentage of correct responses as function of noise level. Blue diamonds represents P(C) when noise is applied only in the stimulator that contains rectangular pulse signal. Red squares represents P(C) when noise is applied on four stimulators simultaneously.

Fig 6 shows the stochastic resonance effect, as noise level increases P(C) also does it, up to certain noise level from which PC) decreases. Can be noted that 1-point noise condition produces greater stochastic resonance than 4-point noise condition. This result was observed in all participants.

#### Experiment 3

**Fig 7:**
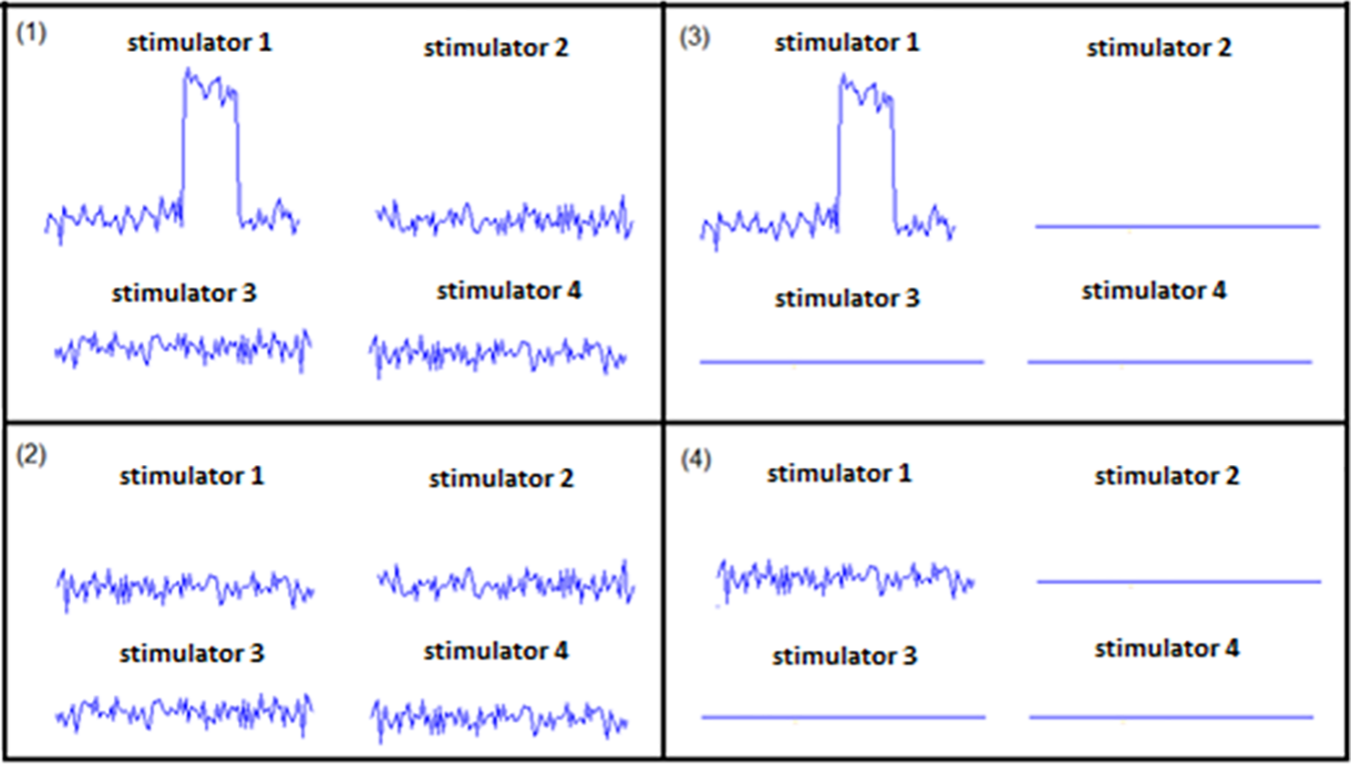
Actual signals captured under four different stimulus conditions. (1) C1: pulse in one stimulator; noise in four stimulators, (2) C2: no pulse; noise in four stimulators, (3) C3: pulse in one stimulator; noise only on the stimulator that contains the pulse, (4) C4: no pulse; noise in one stimulator only.

This experiment was performed to assess the effect of the spatial location of the noise on detection, using two different noise spatial distributions. As shown in Fig 7, there were four different stimuli conditions presented to the subjects. Two of them, conditions C1 and C2, had noise in all the stimulators while the other two, conditions C3 and C4, had noise in only one of them. Conditions C1 and C3 contained the rectangular pulse stimuli, which had an amplitude that was 80% of the measured pulse threshold, and duration of 300ms randomly allocated within the 1.5s attention interval. The spatial position of the stimulus signal was also randomly selected for each trial. Each noise signal lasted 1.5s. Note that for stimulus condition C3, the noise was on the same stimulator that contained the rectangular pulse signal and was, therefore, spatially coincident with the stimulus signal, while in condition C4 there was noise in only one stimulator and no stimulus. Noise stimuli level was applied at their optimal as determined in Experiment 2, and noise stimuli at different skin locations were uncorrelated. A yes/no task[20] was performed with 28 trials of each condition (C1, C2, C3 and C4) randomly presented to observer, giving a total of 112 trials, the percentage of correct responses, P(C), was computed for each condition.

## Results and discussion

### Experiment outcomes

For each subject, the entire experiment was performed three times on three different days, so for each parameter (noise thresholds, pulse threshold, optimal noise level and positive detection rates) were three measurements.

#### Experiment 1 outcomes

**Fig 8:**
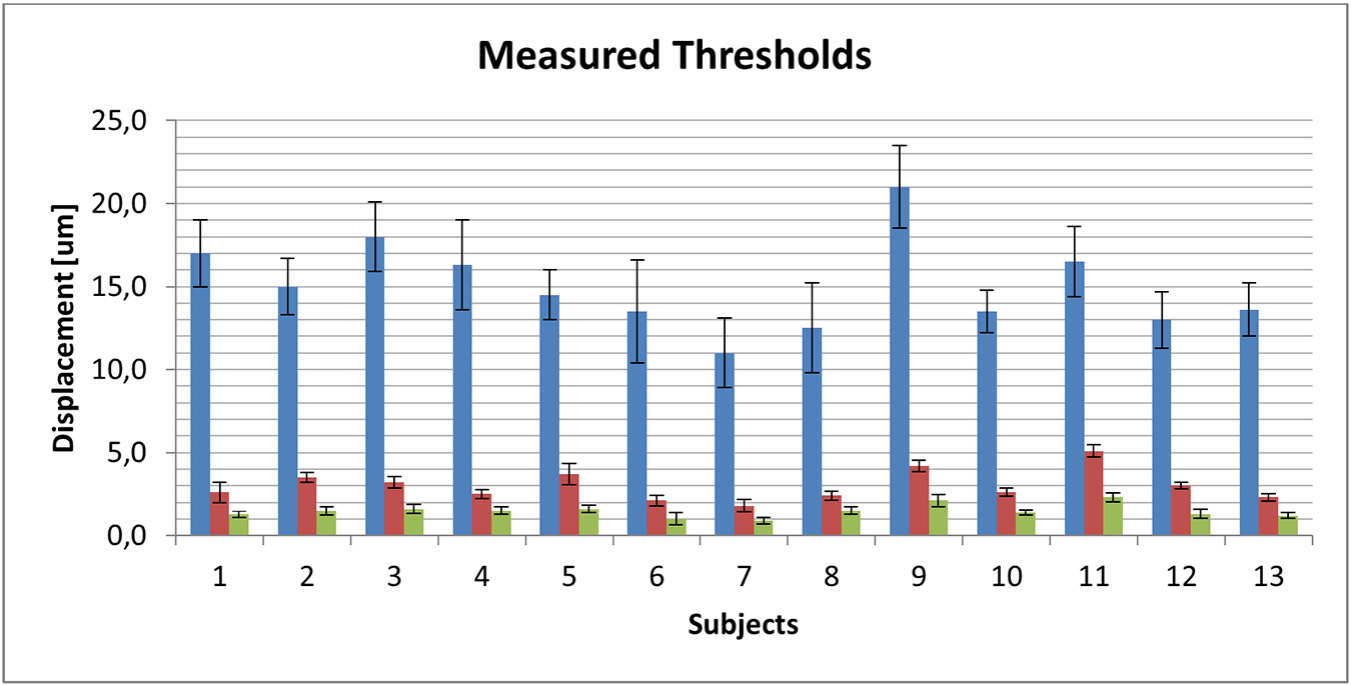
Measured mean values of tactile perception thresholds in micrometers. Blue bars represent measured thresholds for rectangular pulse stimuli. Red bars represent measured thresholds for noise applied in one stimulator. Green bars represent measured thresholds for noise applied in four stimulators. Error bars represent standard deviations over three different measurements for threshold for each subject on three different days.

Tactile perception thresholds were successfully measured for all 13 subjects, to three different stimuli signals: rectangular shape in one stimulator, noise in one stimulator, and noise in all four stimulators. The resulting measured thresholds are shown in Fig 8.

#### Experiment 2 outcomes

**Fig 9:**
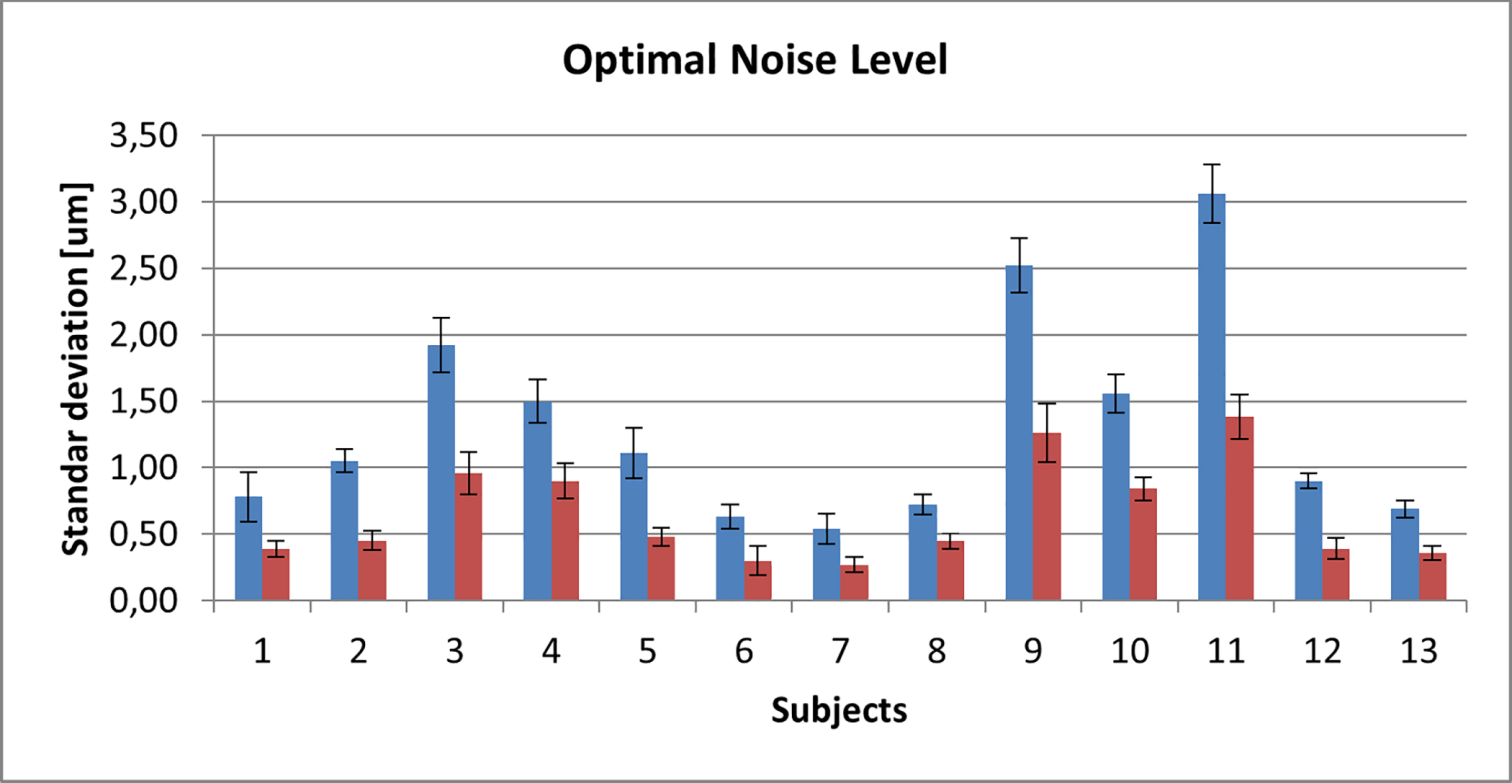
Measured mean values of optimal noise level in micrometers for standard deviation of Gaussian noise. Blue bars represent optimal noise level present only in the same stimulator that contains the rectangular stimuli signal. Red bars represent optimal noise level present in four stimulators simultaneously.

Optimal noise levels which produce stochastic resonance with correct response rates above chance were measured for all 13 subjects, for two different conditions: noise only in the same stimulator that contains the rectangular stimuli and noise in all stimulators simultaneously. The optimal noise levels were determined taking as optimal the level which produced the maximum correct response rate among the 5 level tested for each subject, results are shown in Fig 9.

#### Experiment 3 outcomes

A total of 112 trials were carried out for each subject, 28 for each stimulus configuration. After each trial the subject was asked whether or not the stimuli were perceived, a yes/no task. However if the answer was ‘yes’, the subject was required to identify the position of the stimuli among the four stimulator contact points, 4AFC. We computed the percentage of correct responses, P(C1)+P(C2) for stimuli conditions C1 and C2. We also computed the percentage of correct responses P(C3)+P(C4), for stimuli conditions C3 and C4. For example, a correct response occurred for P(C1) if stimulus condition C1 was presented and the subject’s answer was ‘yes’; for the stimulus condition C2 a correct response occurred for P(C2) if the subject’s answer was ‘no’.

According to SR theory, the added optimal noise in both stimuli conditions C1 and C3 helped the stimulus to exceed the perception threshold above the chance level. SR predicts no difference in sensory performance for conditions C1 and C3, since in both cases threshold crossing was caused by added noise to the original stimulus. As optimal noise level remained lower than the perception threshold, the presence of noise in one or four stimulators produces no difference in correct hit rates. From the point of view of SR, noise is only useful when it helps subthreshold signals reach levels above threshold.

**Fig 10:**
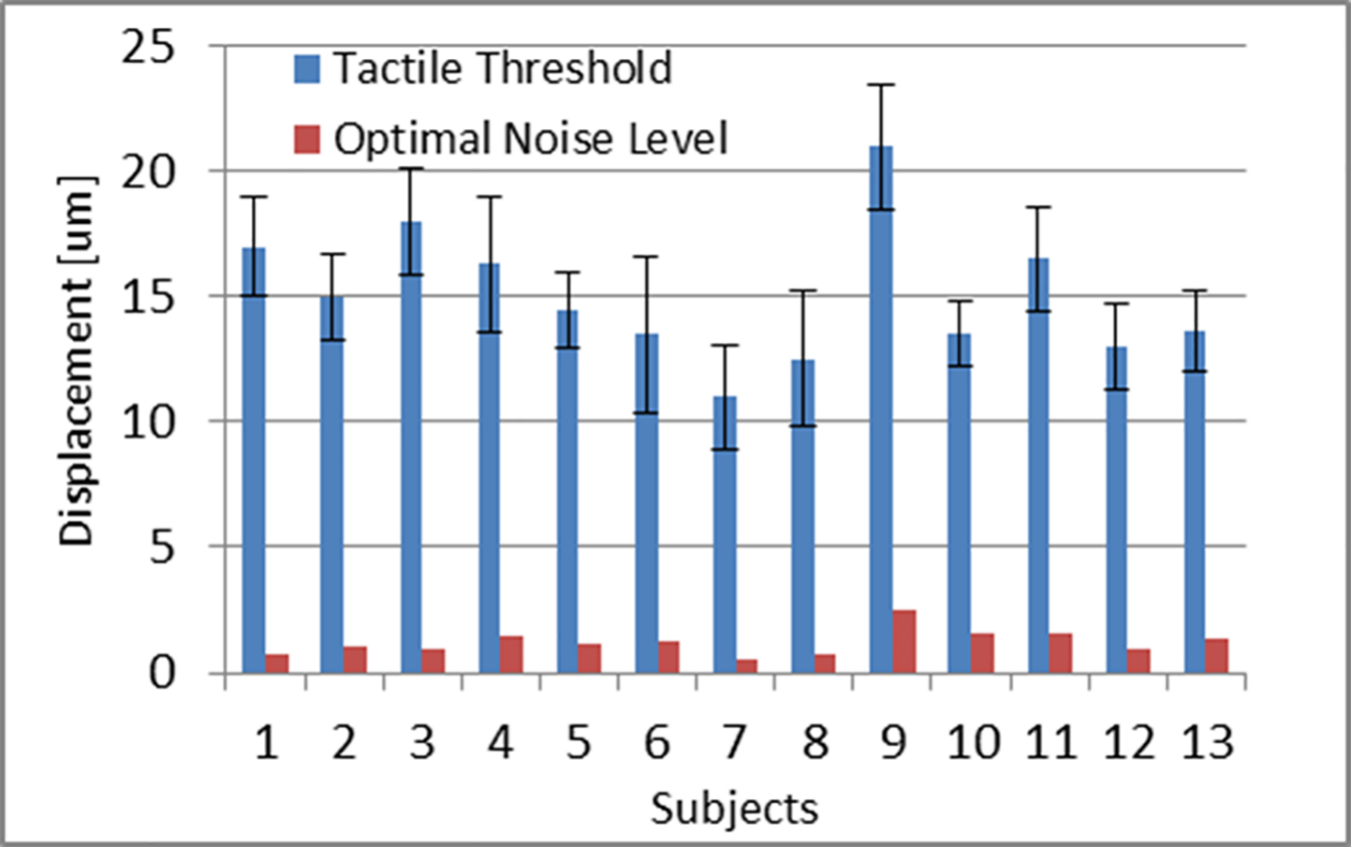
Measured means values for tactile threshold and optimal noise which produce SR, measured as standard deviations. The blue bars indicate the individual threshold level for each subject expressed in micrometers. The red bars represent corresponding one stimulator noise level that produces SR for each subject; noise is expressed in micrometers of the noise standard deviation. Error bars represent standard deviations over three measurements for threshold.

Experiments 1 and 2 were intended to determine the perception thresholds and optimal noise that produces SR. After a series of training sessions, the data was collected from the 13 subjects. Individual values of thresholds and optimal noise levels for the entire group are shown in Fig 10.

The SR behavior [22] was measured for the 13 subjects, i.e., the noise level which maximizes positive detection rates *P*(C) was found above the level of statistical significance (*p*<0.05), where *P*(C) was larger than that expected by chance [2]. Results obtained from Experiments 1 and 2 are comparable to those of previous work [1], [2], [3], [7], [8], [23].

**Fig 11:**
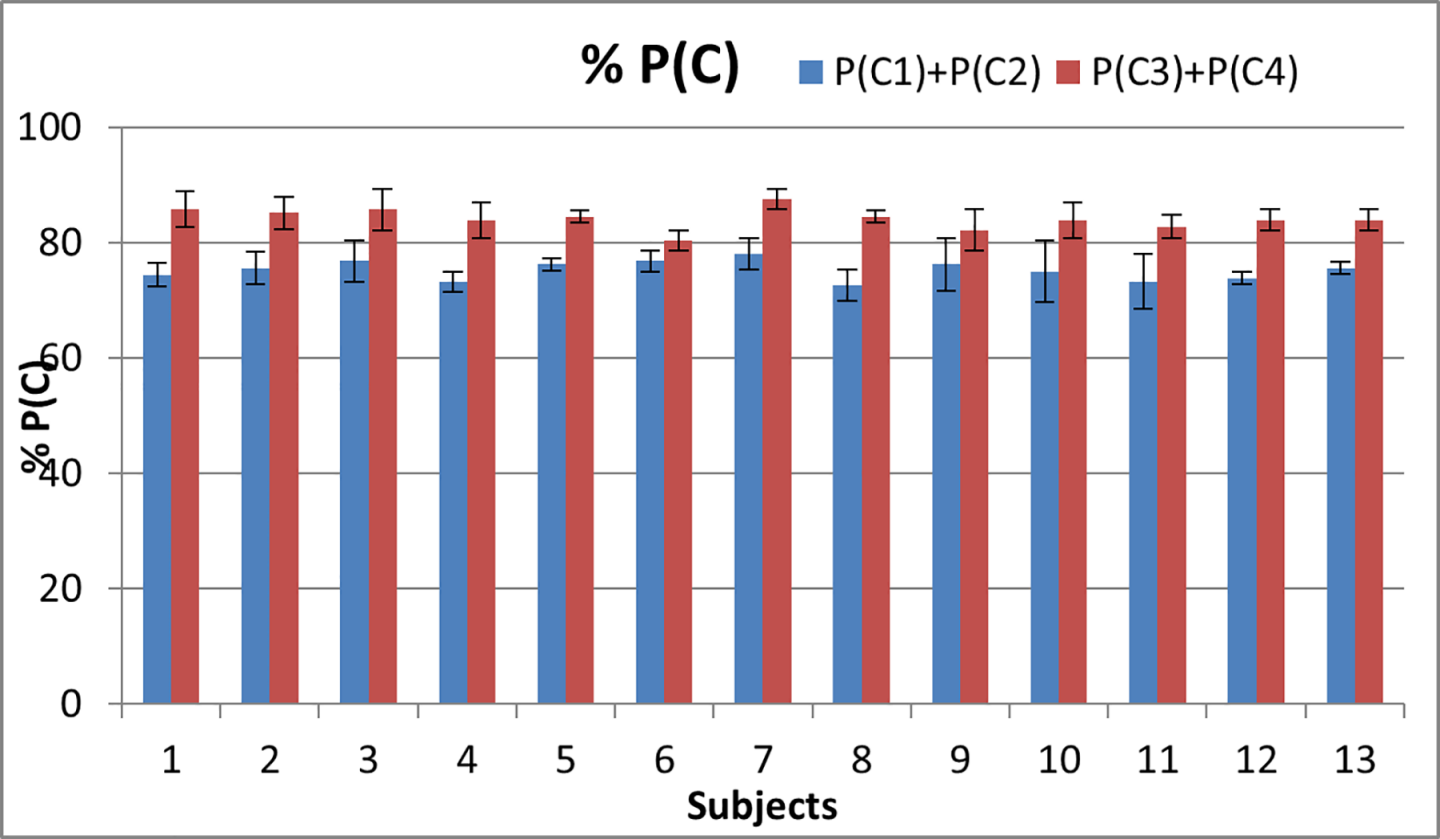
Percentage of correct responses for each subject. Blue bars correspond to P(C1)+P(C2) in which noise is present in all four stimulation points. Red bars correspond to P(C3)+P(C4) where noise is present only at the stimulation point that contains a rectangular pulse.

In Experiment 3, we assessed the effect of the spatial location of the noise within the four stimulation points. For signal condition C1, noise was present in all four stimulation points, while in signal condition C3 noise was present only at the stimulation point that contained the stimulus signal. Correct detection percentage was measured for all the 13 subjects, and as is shown in Fig 11, P(C3)+P(C4) was greater than P(C1)+P(C2) for each subject. An ANOVA test shows that the difference between P(C1)+P(C2) and P(C3)+P(C4) is above chance levels (p<0.001). Therefore, these results show that for each subject the detection rates for the signal conditions C3 and C4, with spatially coincident noise were higher than those for signal conditions C1 and C2 with spatially distributed noise.

Thus, the stimulus with spatially coincident noise enhances detection rates. This is a novel finding that has not been reported previously according to our literature review.

Previously, the *“Coincidence-Enhanced Stochastic Resonance (CESR)”* [1] has been described as the phenomenon in which temporal synchronization of noise with the stimulus can improve response in tactile detection tasks. We propose the term Spatial-Coincidence-Enhanced Stochastic Resonance (SCESR) to describe this novel phenomenon. In the literature we found that, for other human senses, the additional spatial information, such as perceptional hints, effectively reduces spatial uncertainty [23], [24]. Further, according to [1], [14], [24], [25], [26], [27], detection tasks may be improved, as long is additional information intended to reduce the memory.

As an alternative possibility to explain this effect we should consider that for conditions C1 and C2, mechanical noise applied at the stimulators might mechanically spread laterally through the skin and, by superimposing, produce some effect on overall magnitude of skin fluctuations. That interaction might produce an increase or decrease of perceived magnitude of tactile stimulation; let’s consider tactile perception thresholds for noise measured in experiment 1: when noise is applied on four stimulators simultaneously, the threshold is about half than when it’s applied in only one stimulator. Hence we can infer that the effect of stimulating multiple nearby points on finger skin produces an increase in overall perceived magnitude. Now let’s observe the results for experiment 2: for all 13 subjects, a stochastic resonance point was found, that is, there was an optimal noise level that produces correct detection rates above chance, for both noise in one stimulator and for noise on four stimulators; in both cases the optimal noise levels were below perception threshold. For experiment 3, the noise level used for conditions C1 and C2 was the one which produced the higher P(C1) + P(C2) on experiment 2, and for conditions C3 and C4 the noise level was the one which produced the higher P(C3)+P(C4) on experiment 2. But the correct detection rates found on experiment 3 where significantly higher for conditions C3 and C4 than those obtained for conditions C1 and C2. Hence, in opposition to results in experiment 1, from experiment 3 we might infer that the effect of stimulating multiple nearby points on finger skin produces a decrease in overall perceived magnitude. Thus it’s not completely clear if the overall effect of adding noise simultaneously on four nearby stimulation points would produce an increase or decrease on overall perceived magnitude, so further investigation and measurements must be performed to clearly assess this effect.

**Fig 12:**
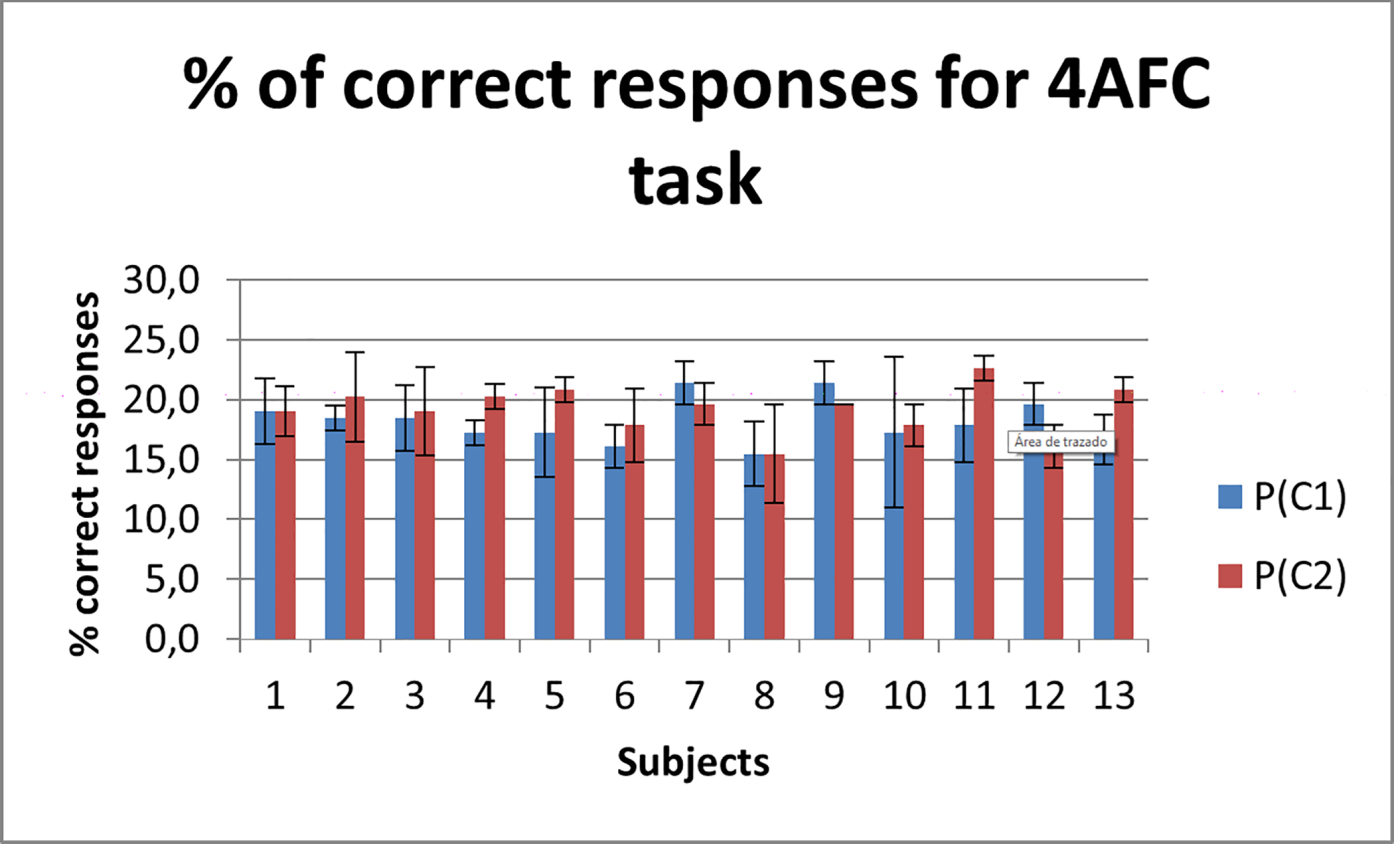
Percentage of correct answers on 4AFC task. Subjects were asked to identify the position where the stimulus was. Blue bars represent the results for condition C1. Red bars represent the results for condition C2. Error bars represent standard deviation over three measurements.

The spatial arrangement of stimulators used on these experiments is similar to tactile sense as the ones used by Cohn in [14] and Phu in [16] who studied the effects of SU on detectability on visual sense; Cohn and Phu found that using signal stimulus that may aid to reduce SU effectively increases detection rates of visual stimuli from different positions on space. Despite using different stimuli source arrangements than this work, all of them, including ours, are selected such as stimulates different receptive fields. However in our experiment subjects were unable to successfully identify the position of the tactile stimuli, so we cannot asseverate that a reduction on SU can be achieved by the methods here exposed. The results of the 4AFC task are shown in Fig 12, where subjects were required to identify the position where the stimulus was.

This effect has never been reported before, and appears to violate standard reports of Stochastic Resonance, and while this is a first report, i.e limited evidence, it remains a novel observation, that needs further corroboration and theoretical interpretation, which we attempted to offer. The effect of putting 4 punctate stimulators in very close proximity to each other still requires an interpretation, but the effect of putting 4 close stimulators together caused the noise perception threshold to be significantly reduced, so it would be logical to think that in that situation there would be more positive detections than when the noise is present in only one stimulator, however the opposite occurred, so the effect of putting the 4 stimulators close to each other is not completely clear.

## Conclusions

New evidence is presented in this work for improvement in the detection of a subthreshold tactile stimulus (Experiment 3 for condition C3) in the presence of spatially coincident noise. We showed that detection improvement is greater when the noise is spatially coincident with the tactile stimulus compared to the situation in which the noise was spatially distributed as in condition C1. Our experimental results showed an interesting phenomenon; while multiple sources of noise increases the overall perceived magnitude when noise perception threshold is measured, the same spatial distribution of noise sources apparently decrease the overall perceived magnitude in detection tasks using tactile stochastic resonance. Additional investigation and experimental data are required to find a possible explanation to this phenomenon.
